# Endothelial Neuropilin Disruption in Mice Causes DiGeorge Syndrome-Like Malformations via Mechanisms Distinct to Those Caused by Loss of Tbx1

**DOI:** 10.1371/journal.pone.0032429

**Published:** 2012-03-02

**Authors:** Jingjing Zhou, Mohammad Pashmforoush, Henry M. Sucov

**Affiliations:** Broad Center for Regenerative Medicine and Stem Cell Research, University of Southern California Keck School of Medicine, Los Angeles, California, United States of America; University of Frankfurt - University Hospital Frankfurt, Germany

## Abstract

The spectrum of human congenital malformations known as DiGeorge syndrome (DGS) is replicated in mice by mutation of *Tbx1*. *Vegfa* has been proposed as a modifier of DGS, based in part on the occurrence of comparable phenotypes in *Tbx1* and *Vegfa* mutant mice. Many additional genes have been shown to cause DGS-like phenotypes in mice when mutated; these generally intersect in some manner with *Tbx1*, and therefore impact the same developmental processes in which *Tbx1* itself is involved. In this study, using *Tie2Cre*, we show that endothelial-specific mutation of the gene encoding the VEGFA coreceptor neuropilin-1 (*Nrp1*) also replicates the most prominent terminal phenotypes that typify DGS. However, the developmental etiologies of these defects are fundamentally different from those caused by absence of TBX1. In *Tie2Cre/Nrp1* mutants, initial pharyngeal organization is normal but subsequent pharyngeal organ growth is impaired, second heart field differentiation is normal but cardiac outflow tract cushion organization is distorted, neural crest cell migration is normal, and palatal mesenchyme proliferation is impaired with no change in apoptosis. Our results demonstrate that impairment of VEGF-dependent endothelial pathways leads to a spectrum of DiGeorge syndrome-type malformations, through processes that are distinguishable from those controlled by *Tbx1*.

## Introduction

DiGeorge syndrome (DGS) is a human genetic disorder characterized by a range of cardiac outflow tract (OFT) and great vessel malformations, craniofacial malformations, hypoplasia of the thymus and parathyroid pharyngeal organs, and cognitive impairment [1]. Most cases of DGS are associated with hemizygous microdeletions of chromosome 22q11.2. The gene encoding TBX1, a member of the T-box containing family of transcription factors, is located within this region and haploinsufficiency of this gene appears to be responsible for most of the manifestations of DGS in humans. In mice, heterozygosity of *Tbx1* can result in impaired 4^th^ pharyngeal arch artery development and thereby in type B interrupted aortic arch [2]–[4], one of the prominent cardiovascular phenotypes in human DGS. Complete absence of TBX1 in mice results in a high severity and penetrance of almost all of the phenotypes seen in human DGS, including cardiac outflow and arch artery defects, pharyngeal organ hypoplasia, and craniofacial malformations [3], [5].

A common theme in the development of the organs that are impacted in DGS is their dependence on neural crest cells. However, *Tbx1* is not expressed in and does not function in neural crest cells [6]–[7]. Rather, *Tbx1* functions in pharyngeal ectoderm, mesoderm, or endoderm (depending on the organ system investigated), and acts through a variety of tissue interactions and downstream effectors in different developmental processes. *Tbx1* is expressed in endothelial cells, although mutation of *Tbx1* in vascular endothelium had no consequence in cardiac, pharyngeal, or craniofacial morphogenesis [8]–[9]. *Tbx1* function in endothelium is required for proper lymphatic vessel development [10], although lymphatic defects are not generally thought to contribute to or be part of the clinical spectrum associated with DiGeorge syndrome.

A number of genes cooperate with *Tbx1* and therefore result in some or all DGS phenotypes when mutated in mice. One such gene is *Crkl* [11]–[12], which is located near *Tbx1* and is therefore also deleted in most cases of DGS. Many other genes that are not physically linked to *Tbx1* have been shown to interact functionally or genetically with *Tbx1* in one or more of the developmental processes in which *Tbx1* participates [2], [12]–[22]. Finally, other genes are known to cause some DGS-like phenotypes when mutated in mice, but have not been analyzed to determine if or how they intersect with *Tbx1*.

Mouse embryos lacking VEGF164, the major isoform of the *Vegfa* gene (which is not chromosomally linked to *Tbx1*), exhibit craniofacial, pharyngeal organ, pharyngeal arch artery, and cardiac outflow tract defects and therefore resemble DGS [23]. Among other sites, *Vegfa* is expressed in the myocardium of the heart outflow tract, in pharyngeal endoderm adjacent to the pharyngeal arch arteries, and in the developing thymus and palate [23]–[25], which is consistent with these phenotypes. Based on a number of arguments (see Discussion), *Vegfa* has been proposed to interact genetically with *Tbx1* and thereby function as a modifier of the DiGeorge syndrome [23]. *Vegfa* deficiency is also associated with generally impaired vascular development, specifically in the formation of small and medium caliber vessels [23], [26]–[27], representing a known function of VEGF in promoting angiogenic vessel branching [27]–[28].

Neuropilin-1 (NRP1) is a dual ligand coreceptor, mediating the effects of VEGF164 when coupled to the VEGF receptor FLK1 (VEGFR2), and the effects of members of the Sema3 family when coupled to several plexins [29]. NRP1 can also mediate VEGF signaling in the absence of VEGFR2 in some contexts [30]. Global knockout of *Nrp1* in mice is embryonic lethal around E12.5; cardiovascular defects in these embryos included a general deficiency in small and medium caliber blood vessel formation, altered pharyngeal arch artery formation, and a failure to septate the outflow tract into the ascending aorta and pulmonary trunk [31], all similar to phenotypes seen in *Vegfa* mutants [23]. Conditional *Nrp1* disruption in endothelial cells using *Tie2Cre* [32] allowed survival to full term, and replicated several of the cardiovascular defects seen in the global *Nrp1* knockout, including general peripheral vascular branching deficiency and failure to septate the outflow tract [33].

In this study, we have investigated the developmental role of *Nrp1* function, specifically in endothelial cell lineages, with particular attention to the developmental processes that are impacted in DGS. In addition to the cardiac outflow tract defect (lack of outflow tract septation) that has already been reported [33], we find that *Tie2Cre/Nrp1* mutants have pharyngeal arch artery, pharyngeal organ, and craniofacial defects that collectively resemble those seen in *Vegfa* mutants and in human DGS. However, we find that the developmental basis of each defect in *Tie2Cre/Nrp1* mutants is dissimilar to what occurs in *Tbx1* null embryos. We suggest that defective vascular organization and endothelial cell dysfunction underlie the phenotypes of *Vegfa* and *Nrp1* mutants. More importantly, our results demonstrate a requirement for *Nrp1* function in endothelium that when compromised leads to DiGeorge syndrome-like defects through mechanisms distinct to those associated with *Tbx1* deficiency.

## Results

### Endothelial-specific deletion of *Nrp1* causes DiGeorge syndrome-like defects in mice

**Figure 1:**
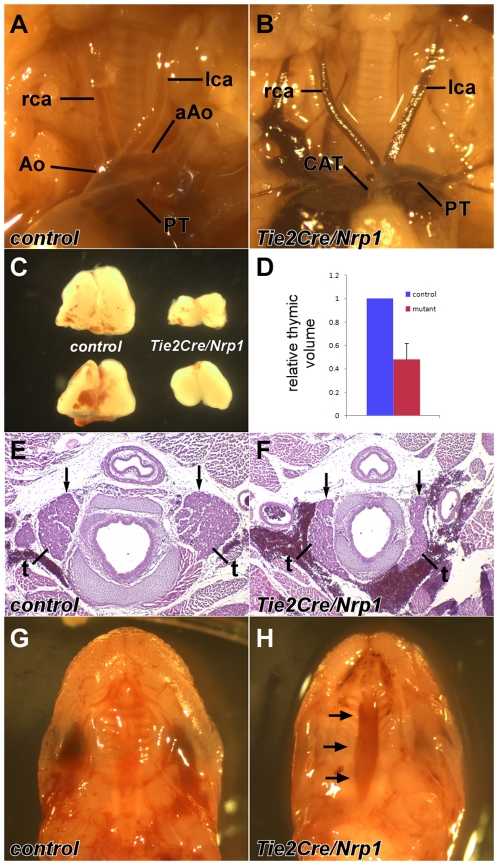
Endothelial-specific deletion of *Nrp1* causes DiGeorge syndrome defects in E18 mice. **A**,**B**, Whole mount views of the outflow region of the heart. The *Tie2Cre/Nrp1* mutant (B) has a common arterial trunk (CAT) and lacks the arch of the aorta (aAo). Other abbreviations: Ao, ascending aorta; lca, left carotid artery; PT, pulmonary trunk; rca, right carotid artery. **C**, Thymuses from two control embryos (at left) and two mutant embryos (at right), all from the same litter. **D**, Quantification of thymic volumes at E18 (n = 6 for each sample). **E**,**F**, Transverse sections showing the thyroid (t) and parathyroid (arrows; the parathyroids are also shown in Fig. S1B–C) in a control (E) and mutant (F). **G**,**H**, Whole mount view of the palate to show clefting (arrows) in the mutant (H).

Embryos with conditional knockout of *Nrp1* driven by *Tie2Cre* survived to full term in normal numbers, but died in the immediate postnatal period. The overall size of E18.5 *Tie2Cre/Nrp1* mutants was not obviously different, and limb development was normal at all stages (Fig. S1A), implying no general embryo-wide growth deficiency or developmental delay. In more than 20 E18.5 mutant embryos, we confirmed that all *Tie2Cre/Nrp1* mutants had a single cardiac outflow vessel (a phenotype called persistent truncus arteriosus or common arterial trunk; Fig. 1A–B), as previously reported [33]. All *Tie2Cre/Nrp1* mutants also had a ventricular septal defect, which is a hemodynamic necessity of a common arterial trunk. In addition, all *Tie2Cre/Nrp1* mutant embryos examined at late stages had some malformation of the great vessels (Fig. 1A–B): a type B interrupted aortic arch was most common, additional great vessel malformations included retroesophageal right subclavian artery and a right sided dorsal aorta. In 10 out of 10 *Tie2Cre/Nrp1* mutants examined at E18.5, the thymus was hypoplastic (Fig. 1C); morphometric quantitation (Fig. 1D) indicated an average 50% decrease in thymic size in mutants relative to littermate controls. Although not quantitated, the thyroid was also hypoplastic in all *Tie2Cre/Nrp1* mutants (Fig. 1E–F), whereas the parathyroids were present and seemingly of proper size (Fig. 1E–F and Fig. S1B–C). Cleft palate occurred in approximately half (9/17) of *Tie2Cre/Nrp1* mutants (Fig. 1G–H); we also observed occasional examples of mandibular hypoplasia (Fig. S1D–E), although in most *Tie2Cre/Nrp1* mutants, the mandible was not obviously dysmorphic. In summation, *Tie2Cre/Nrp1* mutants have a high or complete penetrance of cardiac outflow tract, great vessel, pharyngeal organ, and craniofacial defects that collectively typify DiGeorge syndrome in humans and that are also seen with high or complete penetrance in *Tbx1* and *Vegfa* mutant mice.

*Tie2Cre/Nrp1* mutation also resulted in two additional phenotypes seen in late gestation embryos. There was a disruption in the continuity of the atrial myocardium in all of five *Tie2Cre/Nrp1* mutants examined for this phenotype (Fig. S1F–G), and in one of seven examined mutants, there were mild defects in the structure of the vertebral bodies (Fig. S1H). Comparable atrial and more severe vertebral phenotypes were observed in *Tie2Cre/plexinD1* mutants [34], but have not been reported in *Vegfa* mutants and are not associated with DiGeorge syndrome. These defects therefore are likely to represent defective semaphorin, not VEGF, signaling through NRP1/plexinD1 complexes in endothelial cells.

Global heterozygosity of *Nrp1*, and endothelial-specific conditional *Nrp1* heterozygosity driven by *Tie2Cre*, are both viable and apparently normal (i.e., no malformations were reported for these genotypes) [31], [33]. In our analysis of 14 *Tie2Cre* conditional heterozygous *Nrp1* mutants at E18.5, we did not see any malformations in the cardiac outflow tract, the great vessels, pharyngeal organs, or craniofacial structures.

### Altered endothelial cell organization and outflow tract cushions in *Tie2Cre/Nrp1* mutants

We next explored the morphogenesis of the cardiac outflow tract, the pharyngeal arch arteries and pharyngeal organs, and the palate to determine if the comparable DGS-like phenotypes seen in *Tie2Cre/Nrp1* and *Tbx1* mutants at late embryonic stages share a common underlying developmental and mechanistic basis.

**Figure 2:**
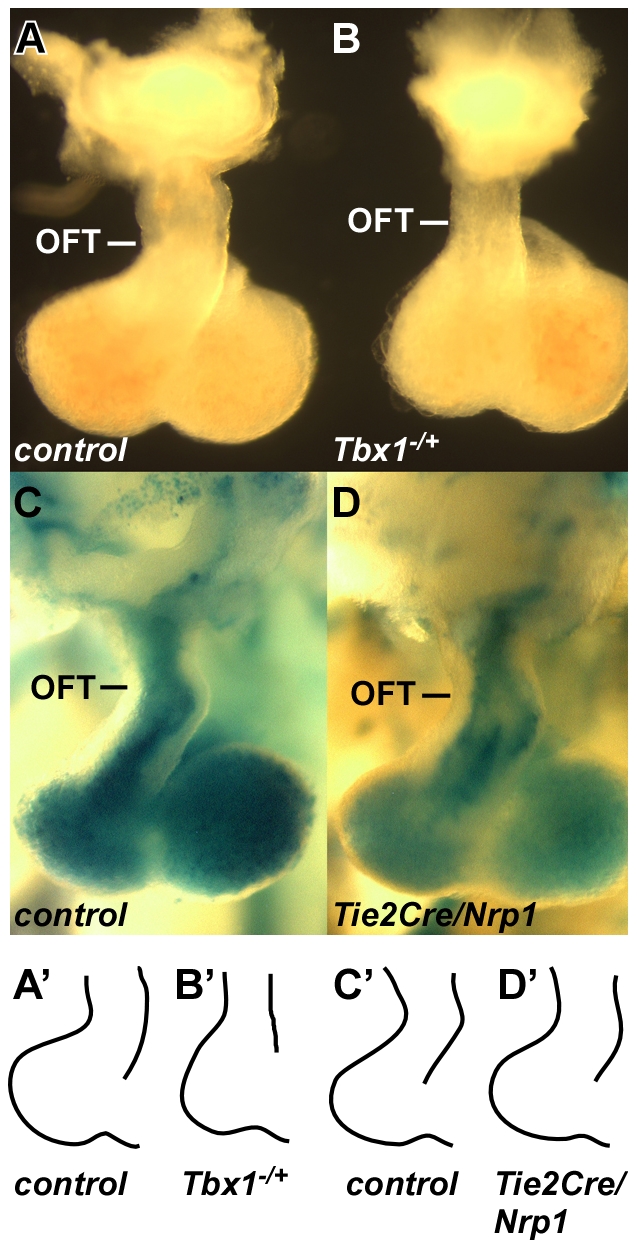
Outflow tract morphology in *Tbx1* and *Tie2Cre/Nrp1* mutants. All hearts shown are from E10.5 embryos. **A**,**B**, control and littermate *Tbx1* null hearts, showing the straight and shortened OFT and the smaller right ventricle in the *Tbx1* mutant. **C**,**D**, control (*Tie2Cre/R26R*) and littermate *Tie2Cre/R26R/Nrp1* mutant, Xgal stained in whole mount, and showing the normal looping of the OFT in *Tie2Cre/Nrp1* mutants. Panels A′,B′,C′,D′ are traced outlines of the same hearts shown in the corresponding panels to more easily compare the absence of outflow tract lengthening and looping in the *Tbx1* mutant to the normal looping in the *Tie2Cre/Nrp1* mutant.

**Figure 3:**
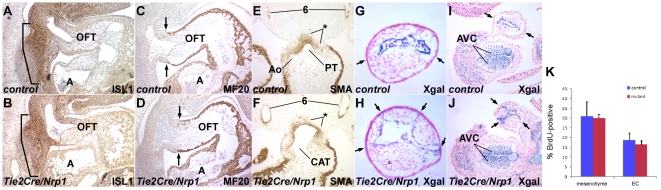
Markers of outflow tract morphogenesis. Upper row in all pairs of panels is a control, lower row is a littermate *Tie2Cre/Nrp1* mutant. **A**,**B**, Immunohistochemical detection of ISL1 at E10.5 (sagittal section); the brackets indicate the ISL1^+^ second heart field. **C**,**D**, Immunohistochemical detection of MF20 at E10.5 (sagittal section); the arrows point to the distal limit of myocardium in the outflow tract. **E**,**F**, Immunohistochemical detection of smooth muscle actin at E11.25 in the outflow tract (transverse section); the asterisks show the location of immunoreactive mesenchyme of presumptive neural crest derivation at the distal end of the outflow tract. In the control, OFT septation is underway although not yet completed, whereas in the mutant, this process fails, resulting in a common arterial trunk (CAT). Positive staining is also apparent in scattered mesenchyme cells lower in the truncal cushions of the outflow tract, and in differentiating smooth muscle surrounding the 6^th^ arch arteries (6). At this stage, SMA also labels myocardium. **G–J**, Orthogonal sections of Xgal-stained *Tie2Cre/R26R* distal outflow tracts from two different control (G,I) and two different *Tie2Cre/R26R/Nrp1* (H,J) E10.5 embryos; the endocardium is Xgal-positive whereas the mesenchyme is unlabeled and therefore of presumptive neural crest derivation. Arrows point to the juxtapositions between the endocardium and the myocardium that separate OFT cushions; there are two such points in controls, and several in mutants indicating abnormal cushion number and organization. Note also the normal extent of Xgal^+^ mesenchyme derived by endocardial transformation in the atrioventricular canal (AVC; I,J). **K**, OFT cell proliferation at E10.5; embryos were BrdU labeled and sections through the distal OFT were immunstained to detect proliferating cells. Mesenchymal cells are of presumptive neural crest derivation, endothelial cells (EC) were identified based on morphology.

*Tbx1* normally functions in pharyngeal and splanchnic mesoderm [8], [35] to maintain the second heart field (SHF), a progenitor population that is added progressively to the distal end of the cardiac outflow tract during the E8–11 period of development [36]. This process allows the OFT to lengthen and thereby achieve a characteristic looped shape by E10.5, and is necessary for septation to occur during the E10.5–11.5 period. In *Tbx1* null embryos at E10.5, as also previously reported [37], the OFT was shortened and poorly looped (Fig. 2A–B). In contrast, the morphology (length and degree of looping) of the OFT was normal in all E10.5 *Tie2Cre/Nrp1* mutants (Fig. 2C–D). ISL1, a marker of the SHF, was normally expressed in *Tie2Cre/Nrp1* mutants (Fig. 3A–B), whereas the domain of ISL1 expression is reduced in *Tbx1* mutants [38]. MF20, a marker of differentiated myocardium, was properly expressed in *Tie2Cre/Nrp1* mutants up to the distal end of the OFT (Fig. 3C–D), whereas MF20 expression extends into the splanchnic mesoderm in *Tbx1* mutants because of premature myocardial differentiation [38]–[39]. These observations imply that the early aspects of SHF differentiation that are controlled by *Tbx1* are normal in *Tie2Cre/Nrp1* mutants.

Neural crest cells are required for OFT septation [40]. NC cells that reach the distal end of the OFT begin to differentiate and express smooth muscle actin (SMA). In *Tbx1* mutants, NC cell migration to the OFT is disrupted [2], [41]. In contrast, we observed a normal number and distribution of SMA-positive cells (of presumptive NC origin) at and around the distal OFT of *Tie2Cre/Nrp1* mutants (Fig. 3E–F). We also crossed the conditional lacZ reporter allele *R26R* into the *Tie2Cre* conditional mutant background and visualized endocardium and endocardium-derived cells in the OFT by Xgal staining. Unstained mesenchymal cells in the OFT are of presumptive NC origin, as the only known sources of OFT mesenchyme are NC and transformed endocardium [32], [42]. The amount and distribution of unstained and stained mesenchyme in the OFT was normal in control and *Tie2Cre/Nrp1* mutants (Fig. 3G–J). Thus, there does not appear to be any apparent defect in NC migration into the OFT that might account for septation failure. Proliferation of mesenchymal neural crest cells and of endothelial cells in the distal OFT was normal (Fig. 3K), and there was also no obvious alteration in endocardium transformation to mesenchyme in the proximal OFT or in the atrioventricular canal (Fig. 3I–J).

Outflow septation involves the growth and fusion of two opposing mesenchymal cushions across the lumen of the OFT [43]. These cushions form by the migration of NC cells between the endocardium and myocardium, and close to the right ventricle, the outflow tract cushions also contain endocardium-derived mesenchyme [42]. In *Tie2Cre/Nrp1* mutants, OFT cushion number and morphology were disorganized. This was seen most clearly in sections orthogonal to the outflow tract (Fig. 3G–J): in normal embryos, the endocardium and myocardium were juxtaposed at two opposite locations (between the two mesenchymal cushions), whereas in mutants, there were multiple sites of association between the endocardium and myocardium (i.e., multiple cushions), and with abnormal attachment of the endocardium to the myocardium.

Valve leaflets form from the distal (truncal) segment of the outflow tract cushions. In normal embryos following septation, both vessels have three valve leaflets. In *Tie2Cre/Nrp1* mutants, we observed a variable number of leaflets (2–4 or more), with these having a range of sizes and morphologies (Fig. S2). Abnormal OFT cushion number and organization therefore might be the initial anatomical defect that leads to improper septation and then also to valve leaflet disorganization.

### Defective arch artery formation in *Tie2Cre/Nrp1* mutants

**Figure 4:**
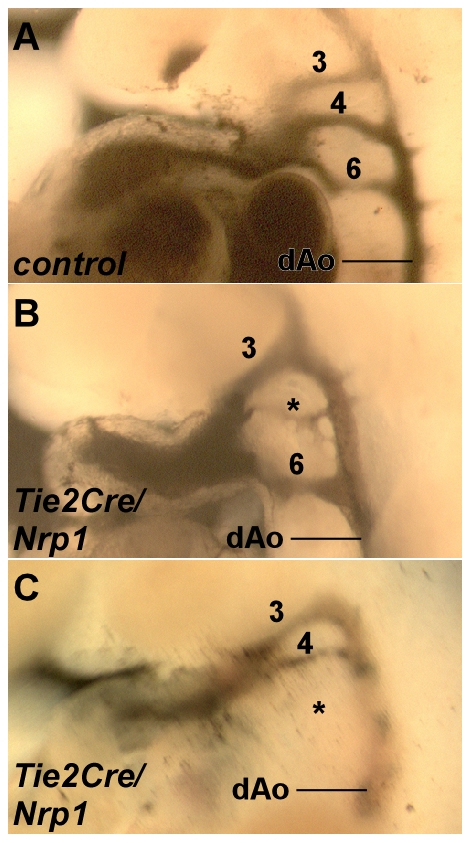
Pharyngeal arch artery organization visualized by ink injection at E10.5. **A**, Normal arterial pattern in a control embryo; the numbers 3, 4, and 6 indicate the respective arch arteries. **B**,**C**, Examples of arch artery abnormalities seen in mutant embryos: an extremely hypoplastic 4^th^ arch artery (B), and a missing 6^th^ arch artery (C), both indicated by asterisks.

The pharyngeal arch arteries form sequentially as the connections between the outflow tract and systemic circulation. At E10.5, the 1^st^ and 2^nd^ arch arteries have regressed and the 3^rd^, 4^th^, and 6^th^ arch arteries are organized in a bilaterally symmetric pattern, connecting to the right and left dorsal aortae. From E11, these arch arteries and segments of the dorsal aortae are reorganized in a highly asymmetric manner to form the mature great vessels [44]. We performed ink injection into the hearts of E10.5 embryos in order to visualize the pattern of arch arteries in control vs. *Tie2Cre/Nrp1* mutants (Fig. 4). The most common defect, seen in 8 of 10 mutant embryos, was a bilateral absence or hypoplasia of both 4^th^ arch arteries (Fig. 4B). In two of these, both 6^th^ arch arteries were also missing, and in one embryo, only the right 6^th^ arch artery was also missing. One mutant had a missing right 6^th^ arch artery with normal 4^th^ arch arteries (Fig. 4C), and one mutant had what appeared to be a normal arch artery organization. These arch artery defects at E10.5 are consistent with the great vessel malformations seen in E18.5 mutant embryos as described above. In *Tie2Cre/Nrp1* mutants, smooth muscle differentiation around arch arteries that were properly formed appeared normal (Fig. 3E–F).

Heterozygosity of *Tbx1* in mice results in a moderate frequency of 4^th^ arch artery defects [2]–[4]. In contrast, no arch artery defects were observed in 13 *Tie2Cre* endothelial-specific conditional *Nrp1* heterozygotes at E10.5 (littermates of those described above), and as noted earlier, no defects were observed in the great vessels or elsewhere in E18.5 conditional *Nrp1* heterozygous embryos. Arch artery defects were also not observed among 24 littermate embryos at E10.5 that lacked the *Tie2Cre* gene.

### Impaired thymic vascular development in *Tie2Cre/Nrp1* mutants

**Figure 5:**
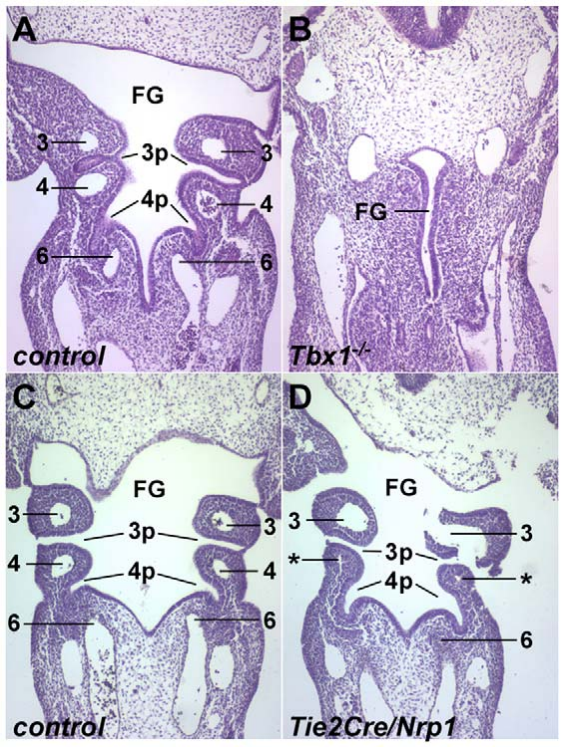
Pharyngeal morphology in *Tbx1* and *Tie2Cre/Nrp1* mutants. All panels are frontal sections of E10.5 embryos. **A**,**B**, control and littermate *Tbx1* null embryos; the *Tbx1* mutant has no segmentation of the foregut (FG) pharyngeal endoderm. **C**,**D**, control and littermate *Tie2Cre/Nrp1* mutant, showing normal pharyngeal organization in mutant embryos even when arch arteries are missing (in the mutant, both 4^th^ arch arteries were extremely hypoplastic, indicated by the asterisks). 3p, 4p, 3^rd^ and 4^th^ pharyngeal pouches; numbers indicate pharyngeal arch arteries. A histology artifact is responsible for the broken piece of the left 3^rd^ arch in the mutant shown.

The thymus and parathyroids are derived from the 3^rd^ pharyngeal pouch. As also previously observed [2], [35], [45], *Tbx1* mutants lacked proper segmentation of the pharyngeal arches and pouches (Fig. 5A–B), which compromises the formation of the thymus and parathyroid organs. In contrast, in every *Tie2Cre/Nrp1* mutant embryo, we observed normal pharyngeal pouch organization, even as pharyngeal arch artery development was obviously compromised (Fig. 5C–D). Thus, the pharyngeal organ defects of E18.5 *Tie2Cre/Nrp1* mutants do not share a common developmental etiology with the similar malformations seen in *Tbx1* mutants. Furthermore, these observations also show that the arch artery and great vessel defects in *Tie2Cre/Nrp1* mutants described above do not occur as the indirect result of pharyngeal disorganization.

**Figure 6:**
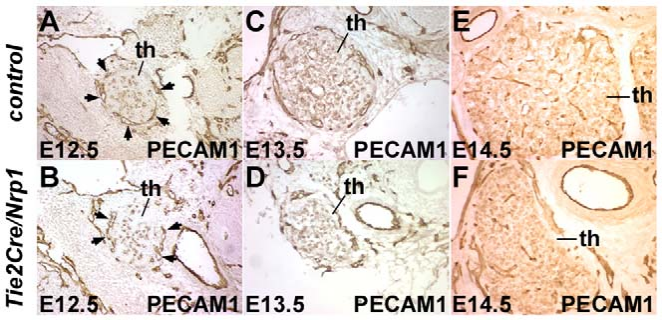
Deficient thymic growth and vascular organization in *Tie2Cre/Nrp1* mutants. Upper row in all pairs of panels is a control, lower row is a littermate *Tie2Cre/Nrp1* mutant. PECAM1 immunostained transverse sections around the developing thymus (th) are shown; all six images are at the same magnification. **A**,**B**, E12.5 embryos; **C**,**D**, E13.5 embryos; **E**,**F**, E14.5 embryos. Arrows in A and B point to nascent vessels at the periphery of the E12.5 thymus, which are similar in control vs. mutant embryos at this stage.

Thymic morphology and size at E12.5 was normal in *Tie2Cre/Nrp1* mutants, but hypoplasia became apparent at E13.5 and persisted proportionally as development proceeded. TUNEL staining showed no difference in apoptosis at any stage (Fig. S3). We used PECAM1 immunostaining to visualize vascular organization in the developing thymus. In normal development, blood vessels initiate assembly around the periphery of the thymus at E12.5 [46], and this occurred properly in mutants (Fig. 6A–B). At E13.5, in control embryos, peripheral vessels were still present and in addition several intrathymic vessels had also formed, whereas in *Tie2Cre/Nrp1* mutants, peripheral vessels were present but with few if any internal vessels (Fig. 6C–D). In control embryos at E14.5, extensive intrathymic vessels were present and now with few peripheral vessels, whereas in mutants, the majority of vessels were still at the thymic perimeter with fewer and less complex intrathymic structures (Fig. 6E–F). Thus, thymic vascular formation is deficient or delayed in *Tie2Cre/Nrp1* mutants.

### Reduced palatal mesenchyme cell proliferation in *Tie2Cre/Nrp1* mutants

**Figure 7:**
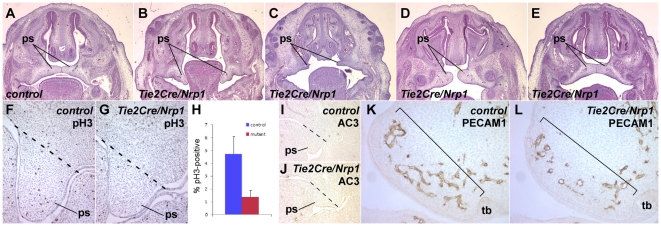
Reduced palatal mesenchyme cell proliferation in *Tie2Cre/Nrp1* mutants. **A–E**, frontal sections at E14.5 of control (A) and of several mutant embryos (B–E) showing a range of palatal phenotypes: failure of the palatal shelves (ps) to elevate (B), gross and mild failure to properly extend (C and D, respectively), and normal fusion (E; approx. half of mutants had normally fused palates). **F**,**G**, Proliferation detected by phospho-histone H3 immunohistochemistry in frontal sections of control (F) and *Tie2Cre/Nrp1* (G) embryos at E13.5 (nuclei are counterstained with hematoxylin); the dotted line shows the approximate upper end of the palatal shelves for purposes of quantification of proliferating cells. **H**, Quantification of mesenchymal proliferation in E13.5 palatal shelves. **I**,**J**, Absence of palatal shelf apoptosis at E13.5 in frontal sections, visualized by immunostaining for activated caspase 3. **K**,**L**, Immunohistochemical detection of PECAM1 in control (K) and mutant (L) embryos (frontal sections) in the palatal shelves at E12.5. The bracket indicates the region of growth and extension; there are fewer and less well elaborated vessels in this region in the mutant. The avascular region behind the brackets in both panels is condensed mesenchyme that will form the maxillae. tb, tooth bud.

The palate forms from opposed shelves in the oral cavity that first elevate, grow towards each other, and then fuse. In normal development, the palatal shelves contact each other at E14.5 and by subsequent breakdown of the medial epithelium become fused at E15.5 [47]. Cleft palate was present in approximately half of E18.5 *Tie2Cre/Nrp1* mutants (described above). We examined younger embryos to determine the basis of this phenotype. At E14.5, we observed a spectrum of palatal shelf hypoplasia, ranging from failure to elevate to various degrees of incomplete growth (Fig. 7A–D). We also observed fusing palatal shelves in approximately half of examined mutant embryos at E14.5 (Fig. 7E), which is consistent with the range of phenotypes scored at E18.5.

In *Tbx1* nulls, cleft palate is a highly penetrant phenotype, and analysis at E14.5 showed a diminishment of palate shelf size [48], similar to what we observed in approximately half of the *Tie2Cre/Nrp1* mutants. In *Tbx1* nulls, there is an increase in palatal mesenchyme cell proliferation that is more than counterbalanced by an increased level of apoptosis, resulting in insufficient net growth and thereby in clefting [48]. In contrast, a similar analysis of *Tie2Cre/Nrp1* mutants revealed a significant decrease in cell proliferation (Fig. 7F–H) and no change in apoptosis (Fig. 7I–J). Thus, the cellular basis of cleft palate in the two models is distinct.

The mesenchyme of the palatal shelves is derived almost completely from neural crest, so a decrease in palatal mesenchyme cell proliferation must be an indirect consequence of *Nrp1* disruption in endothelium. We used PECAM1 immunostaining to visualize the developing vasculature in the palatal shelves. At E12.5, we consistently observed fewer blood vessels in mutants compared to controls (Fig. 7K–L). This may be the initial defect that leads to decreased mesenchymal cell proliferation and thereby to cleft palate. At E13.5 and E14.5, vascular distribution was more variable in mutants and often resembled controls; this may explain why palate fusion was achieved in approximately half of mutant embryos by E14.5.

### Lack of genetic interaction between *Nrp1* and *Tbx1*

In mice, the primary phenotype in *Tbx1* heterozygotes is a variable penetrance of 4^th^ arch artery hypoplasia (resulting in type B interrupted aortic arch in later embryos), although the penetrance of this phenotype is strain-dependent. All other DGS defects occur when *Tbx1* is homozygous, or when *Tbx1* heterozygosity is combined with mutation of another interacting gene. We addressed whether heterozygous *Nrp1* deficiency in the *Tie2Cre* domain synergized with global heterozygous *Tbx1* deficiency. In the outbred genetic strain background of our mice, there was no incidence among 9 *Tbx1* heterozygous embryos analyzed of interrupted aortic arch nor of any other malformation. All of 7 *Tie2Cre,Nrp1^fl/+^,Tbx1^−/+^* embryos, analyzed at E18, were also normal in all regards (Fig. S4), implying no detectable enhancement of phenotypic penetrance. The power of this analysis is limited by the absence of observed phenotypes, and detection of a possible epistatic interaction might require backcrossing onto a more susceptible genetic background. However, the apparent lack of genetic interaction is fully consistent with the observations described above showing a different cellular and developmental basis for each DGS phenotype in *Tie2Cre/Nrp1* and *Tbx1* mutants. Collectively, our results imply that *Tbx1* and *Nrp1* are parts of distinct pathways that lead to DGS malformations.

## Discussion

In this study, we report that endothelium-specific disruption of *Nrp1* causes DiGeorge syndrome-type malformations in mice. An unseptated outflow tract (common arterial trunk) was noted in an earlier description of *Tie2Cre/Nrp1* mutants [33], and global *Nrp1* disruption was previously shown to result in early pharyngeal arch artery defects [31] that would be manifest as great vessel malformations if such embryos were able to survive to late gestation. However, the constellation of cardiovascular outflow tract, great vessel, pharyngeal organ, and craniofacial defects that collectively typify DiGeorge syndrome had not been previously recognized in *Tie2Cre/Nrp1* mutants, and could not have been seen (because of early lethality) in global *Nrp1* mutants.

Because NRP1 functions as a coreceptor with VEGFR2 for VEGF signals, and with plexins for Sema3 signals, *Nrp1* mutant phenotypes might reflect either or both signaling pathways. Cleft palate or pharyngeal organ malformations are not known to result from plexin or semaphorin gene mutations, but do occur in *Vegfa* mutants, implying that these phenotypes in *Tie2Cre/Nrp1* mutants represent *Vegfa* signaling pathways. Cardiac outflow tract defects are observed in *Sema3c* and in *plexinD1* mutants as well as in *Vegfa* mutants; however, a previous detailed genetic analysis showed that NRP1 is required for VEGF but not for Sema3 signaling in this tissue [33], [49]. We conclude therefore that NRP1 is required as a receptor for VEGF signals that are important in cardiac outflow tract, arch artery, pharyngeal organ, and craniofacial development (i.e., the organs impacted in DiGeorge syndrome). In contrast, the atrial myocardium and vertebral defects seen in *Tie2Cre/Nrp1* mutants are not known to occur in *Vegfa* mutants but are manifest in *Tie2Cre/plexinD1* mutants, implying that NRP1 serves as a plexinD1 coreceptor for Sema3 signals that support the integrity of the atrial myocardium and that mediate vertebral skeletal organization.

One of the best known roles of VEGF is to support blood vessel vasculogenesis and angiogenesis, and previous descriptions of *Vegfa* [23], [26]–[27] and *Nrp1* [31], [33] mutants reported a deficiency of blood vessel density, specifically of medium and small caliber vessels, in tissues throughout the embryo. We observed the same type of deficiency in the palate and thymus of *Tie2Cre/Nrp1* mutants, which may underlie the diminished growth of these organs during mid- to late gestation. The great vessel defects seen in this mutant background result from a failure of some of the initial arch arteries to form. *Vegfa* is expressed in pharyngeal endoderm [23]–[24], and is therefore in close proximity to influence the behavior of NRP1-expressing endothelial cells in the pharyngeal arches. We do not yet know if the primary defect is in the initial organization of endothelial cells into nascent vessels, or in their subsequent interaction with neural crest cells that is required for vessel stabilization [50].

The outflow tract phenotype of *Tie2Cre/Nrp1* mutants appears to be qualitatively distinct from the vascular defects seen elsewhere in these embryos. *Vegfa* is expressed in outflow tract myocardium [23]–[25], and our results suggest that it signals through NRP1 in the endocardium to facilitate cushion organization, which is required for subsequent septation and valve formation. Distortion of OFT cushion organization is likely to be the underlying cause of the single persisting outflow vessel in *Tie2Cre/Nrp1* mutants, as the ultimate fusion of these malformed and mispositioned cushions would result in the obstruction of one of the two outflow channels [51].

DiGeorge syndrome in humans is most commonly associated with mutation of the *Tbx1* gene, and in mice, the terminal DGS phenotypes of *Tie2Cre/Nrp1* and *Tbx1* mutants are similar. Nonetheless, our analysis revealed fundamental differences in the developmental process that lead to these phenotypes in the two mutant backgrounds. Thus, in *Tbx1* mutants, a second heart field deficiency leads to a shortened and misaligned outflow tract [8], [35], [37] (Fig. S2A–B), defective neural crest cell migration and/or smooth muscle differentiation appear to be responsible for arch artery defects [2], [7], [41], [52], disorganization and lack of segmentation of the pharyngeal endoderm results in pharyngeal organ defects [2], [35], [45] (Fig. 5A–B), and elevated mesenchymal apoptosis leads to cleft palate [48]. None of these events occur in *Tie2Cre/Nrp1* mutants. Instead, we have described alternate developmental processes that explain the various phenotypes. If *Nrp1* and *Tbx1* converge in a functionally relevant manner, we would predict not only similar terminal mutant phenotypes but also similar intermediate developmental manifestations, which we did not see. Our results lead to the conclusion that a broad range of DGS phenotypes can occur as the result of disruption of a single gene (*Nrp1*) and through mechanisms that are distinct from those associated with *Tbx1*.

We believe that our conclusion of a *Tbx1*-independent pathway leading to the spectrum of defects that typify DGS is novel. Many mouse gene mutations result in a similar range of DGS-type malformations, but these genes have either been shown to interact genetically or mechanistically with *Tbx1* and therefore impair the same developmental processes as does *Tbx1*, or simply have not been examined in detail. Many other gene mutations cause individual phenotypes but not the larger constellation of DGS malformations, and these clearly need not intersect with *Tbx1*. It is also noteworthy that no previous genetic manipulation that results in the broad spectrum of DGS-type defects has been shown to involve the endothelium, and in the case of *Tbx1*, an endothelial role has been disproven [8]–[10]. One possible example similar to *Nrp1* involves the endothelin signaling system, where mutation of the ligand gene *Edn1* [53]–[54], of the receptor gene *Ednra* [55], or of the converting enzyme gene *Ece1* [56]–[57] all lead to DGS-type malformations. Analysis of *Tbx1*-*Ece1* trans heterozygotes failed to reveal genetic interaction [58], as we also observed between *Tbx1* and *Nrp1*. However, if endothelin is part of the VEGFA-NRP1 axis rather than part of the TBX1 pathway, the relationship must be indirect: *Ednra* function in neural crest cells is required for the normal morphogenesis of the cardiac outflow tract, arch arteries, pharyngeal organs, and craniofacial structures [59], although endothelial-specific mutation of *Edn1* caused no developmental malformations [60].

*Vegfa* has been proposed as a genetic modifier of the chromosome 22q11 deletion syndrome [23], based on several criteria: the occurrence of similar phenotypes in mouse *Vegfa* mutants as in *Tbx1* mutants, the observation of genetic synergy between *vegfa* and *tbx1* in zebrafish embryos specifically for arch artery defects, a *Vegfa* haplotype in humans that was associated with increased incidence of cardiovascular phenotypes in chromosome 22q11 patients, and finally by the decreased expression of *Tbx1* in the pharyngeal region of E10.5 *Vegfa* mutant mouse embryos. *Tie2Cre/Nrp1* and *Vegfa* mouse mutants share most DGS-related phenotypes, yet our results imply that *Tbx1* is not involved in the etiology of any of the *Tie2Cre/Nrp1* defects. Thus, it may also not be necessary to invoke *Tbx1* to explain most of the *Vegfa* deficient phenotypes. A prior study also questioned whether non-*Tbx1*-dependent mechanisms explain the effects of VEGF on pharyngeal arch artery development [9]. At the same time, in some tissues that are outside of the *Tie2Cre* recombination domain, VEGF may also regulate or intersect with *Tbx1*. This may for example explain the reported decrease in *Tbx1* expression in the pharyngeal arch region of *Vegfa* mutants, and the higher incidence and severity of mandible hypoplasia in *Vegfa* mutants compared to *Tie2Cre/Nrp1* mutants.

With the exception of the outflow tract phenotype, the other VEGF-dependent defects observed in *Tie2Cre/Nrp1* mutants appear to reflect deficient vascular formation (great vessel defects) or angiogenesis (for the thymus and palate). Deficient vascular organization has previously been associated with teratogen-induced and spontaneous cleft palate [61]–[62], and a “vascular hypothesis” has been proposed to more broadly explain DiGeorge syndrome phenotypes as the consequences of vascular insufficiency [63]. Clearly in the case of chromosome 22q11 deletion syndrome and for *Tbx1* mutation, a disruption of earlier processes is a more suitable explanation for these defects. However, our results suggest that VEGF- and NRP1-dependent vascular defects can result in many DGS phenotypes in a manner that is independent of and later in developmental time than when the *Tbx1* gene functions, at least as *Tbx1* function is currently understood.

There is an alternative interpretation of our observations and of those from past studies that we cannot formally exclude. TBX1 clearly controls many early developmental processes in an NRP1-independent manner that when compromised result in DGS. However, *Tbx1* might in addition also control VEGF signaling to endothelial NRP1 in a way that accounts for the later alternative defects that we observed in *Tie2Cre/Nrp1* mutant embryos. The lack of genetic interaction between *Tbx1* and *Nrp1* argues against this model, although the negative result does not disprove its possibility. If this model is true, *Tbx1* controls two independent sets of processes (earlier NRP1-independent and later VEGF/NRP1-dependent) that both lead to the same spectrum of DGS phenotypes. This would be a novel aspect of *Tbx1* function that has been obscured by all of the early (NRP1-independent) defects that alone are sufficient to explain DGS phenotypes in *Tbx1*-null embryos.

In *Tie2Cre/Nrp1* mutants, deficient vascular organization is not confined to only the cardiac outflow tract, pharyngeal arch arteries, pharyngeal organs, and palate; similar vascular deficiencies occur throughout the embryo [33], including many organs that are not grossly impacted by *Nrp1* mutation and that are not part of the DGS spectrum. This is also true for *Vegfa* mutation [23], [26]–[27]. VEGF signaling through endothelial NRP1 is therefore responsible for local vasculogenesis and angiogenesis, although most tissues are not apparently affected when this process is compromised. The unexpected observation is that the tissues that are most commonly impacted in DiGeorge syndrome appear to be particularly dependent on VEGF-NRP1 signaling. It has long been appreciated that neural crest cells contribute to the tissues that are compromised in DGS. Thus, NC cells are required for OFT septation [40], support the formation of the pharyngeal arch arteries [50], form the pericytes and smooth muscle of the thymic vasculature [64]–[66], and contribute virtually all of the mesenchyme that allows growth of the palatal shelves [47]. Moreover, ablation of the NC can phenocopy DGS [67]. We speculate that a VEGF-dependent interaction between endothelial and neural crest cells may account for the restricted (DGS spectrum) distribution of phenotypic consequences in *Tie2Cre/Nrp1* and *Vegfa* mutants. For arch artery formation and cardiac outflow tract septation, this might involve a direct physical or signaling interaction between VEGF-activated endothelial cells and neural crest cells. Alternatively, neural crest cells are thought to be particularly sensitive to hypoxia [68]–[69], and organs such as the thymus or palate that depend on NC cells for their growth might be indirectly compromised as the consequence of vascular deficiency.

Most cases of DGS in humans are associated with *Tbx1* deficiency; however, a number of DGS patients lack chromosome 22 deletions and do not appear to have mutations in the *Tbx1* gene [70]. A second DGS locus has been postulated to reside on human chromosome 10 [71]–[72], in the vicinity of the *Nrp1* gene. *Nrp1* may therefore be a candidate gene to explain DGS phenotypes in patients without *Tbx1* mutations.

## Materials and Methods

### Mice

*Tie2Cre* [32], *Neuropilin-1^flox/flox^* [33], *Tbx1^−/+^* [3], and *R26R* [73] mice were bred according to standard protocols. All animal work was conducted according to relevant national and international guidelines. Full details of this study were reviewed and approved by the USC IACUC (protocol #10848).

### Immunohistochemistry

Embryos were fixed in 4% PFA overnight at 4°C, then dehydrated in an ethanol series and embedded in paraffin, and sectioned at 10 µ thickness. Following dewaxing and rehydration, primary antibodies used were against MF20 (Developmental Studies Hybridoma Bank; 1∶20 dilution), ISL1 (Developmental Studies Hybridoma Bank; 1∶20), and SMA (Sigma, 1∶1000). For BrdU detection, pregnant females were injected with 100 µg/g body weight BrdU 2 hr before sacrifice, followed by fixation, paraffin embedding, and sectioning as above; primary antibody was from the BrdU Staining kit (Invitrogen). For detection of CD31 (BD Pharmingen MEC13.3; 1∶400) and phospho-histone H3 (Cell Signaling; 1∶100), embryos were fixed as above then transferred to 10% and then 30% sucrose, then embedded in OCT on dry ice, and 10 µ frozen sections were cut. Biotin-conjugated secondary antibodies (Santa Cruz, 1∶300) were used according to the primary antibody species. Streptavidin-HRP was applied to slides after secondary antibody incubation; signal was detected with the DAB Plus kit (Invitrogen). In general, sections were counterstained with hematoxylin prior to mounting.

### In situ hybridization

Embryos were isolated in RNase free PBS on ice. The isolated embryos were fixed in 4% PFA overnight at 4°C, then transferred to 10% and then 30% sucrose, then embedded in OCT on dry ice. 10 µ sections were cut. Digoxygenin-labeled PTH probe was made by in vitro transcription from a template obtained from N. Manley. Signals were detected with anti-digoxygenin alkaline phosphatase-coupled antibody (Roche) and BM Purple substrate kit (Roche).

### Ink injection

Embryos were isolated and kept in PBS on ice. The body wall was opened and the right ventricle exposed; India ink (Martin's Bombay Black, diluted in PBS as needed) was injected directly into the right ventricle by mouth pipetting with a drawn out glass micropipet. Embryos were then fixed in 4% PFA overnight.

### X-gal staining

Embryos were isolated on ice and fixed with 0.2% glutaraldehyde at 4°C for 10 min, then whole mount stained in Xgal at room temperature overnight. For sections, stained embryos were processed to 10% and 30% sucrose, then embedded and frozen in OCT. 10 µ sections were rinsed in PBS and counterstained with nuclear fast red.

### TUNEL assay

Paraffin sections were cut at 10 µ; the remaining procedures were done according to the In Situ Death Detection kit protocol (Roche). The positive controls were prepared from sections of the same embryos by preincubation with DNaseI to induce DNA strand breaks, according to the kit protocol.

### Bone and cartilage staining

E18.5 embryos were isolated and fixed in 95% ethanol overnight. Following a brief rinse in deionized water, cartilage was stained with alcian blue (0.05% alcian blue 8GX/5% acetic acid in distilled water) for 24 hours. Stained embryos were washed in 70% ethanol for 6–8 hr, then transferred to 1% potassium hydroxide overnight. Bone was stained with alizarin red (0.005% (w/v) in 1% KOH) for 2–4 hr. The tissue was then cleared in 1% KOH/20% glycerol for 2 or more days.

### OFT valve staining

The OFT valve region of E18.5 embryos was dissected manually, then fixed in 4% PFA overnight. The tissue was whole mount stained with alcian blue (0.05% alcian blue 8GX/5% acetic acid in distilled water) for 24 hr, then rinsed in water and photographed using darkfield optics.

## Supporting Information

Figure S1 **Embryogenesis in** ***Tie2Cre/Nrp1*** **mutants.** **A**, Comparison of E14.5 forelimbs from a control (top) and *Tie2Cre/Nrp1* mutant (below) littermates to show normal limb development. **B**,**C**, In situ hybridization detection of parathyroid hormone to visualize the parathyroids in a control (A) and mutant (B) embryo at E18. **D**,**E**, Images of E14.5 heads of control (C) and mutant (D) embryos; the arrowhead in E points to the one obvious example of mandibular hypoplasia seen in this mutant background in this study. The shiny appearance of the embryo in panel E is a lighting artifact. **F**,**G**, Atrial myocardium at E14.5 in a control (F) and mutant (G) embryo; in the mutant, the myocardium is detached from the epicardium and is split into multiple pieces (two shown by arrows). **H**, Skeletal preparation of one mutant embryo at E18.5 showing vertebral defects, specifically an incompletely formed lumbar L2 vertebral body, and a partial fusion of the L7 and L8 vertebrae.
(TIF)

Figure S2 **Visualization of valve leaflets at E18.5.** **A**, **A′**, A single control embryo shown from two slightly different angles to visualize the aortic and pulmonary valves, each consisting of three well-formed leaflets. **B–E**, Four different mutants all showing a single outflow structure (i.e., a common arterial trunk) with a variety of valve leaflet morphologies and organizations.
(TIF)

Figure S3 **Absence of apoptosis in the developing thymus.** **A**,**B**, Sections of E14.5 embryos stained by TUNEL labeling. Nuclei are counterstained in blue, and TUNEL-positive cells are in green; there were virtually no positive cells in either genotype. **C**, A positive control for staining is shown at the same magnification.
(TIF)

Figure S4 **No accentuated phenotypes in** ***Tbx1*** **-** ***Nrp1*** **trans heterozygotes, all analyzed at newborn stage.** **A**,**B**, Palates were fully closed. **C**, Heart outflow septation and great vessels were normal. **D**, Thymic size was normal.
(TIF)
