# Supplementary figures and images for: Endothelial Neuropilin Disruption in Mice Causes DiGeorge Syndrome-Like Malformations via Mechanisms Distinct to Those Caused by Loss of Tbx1
Source: PLoS One. 2012 Mar 2;7(3):e32429. doi: 10.1371/journal.pone.0032429 (PMC3292556; doi:10.1371/journal.pone.0032429)

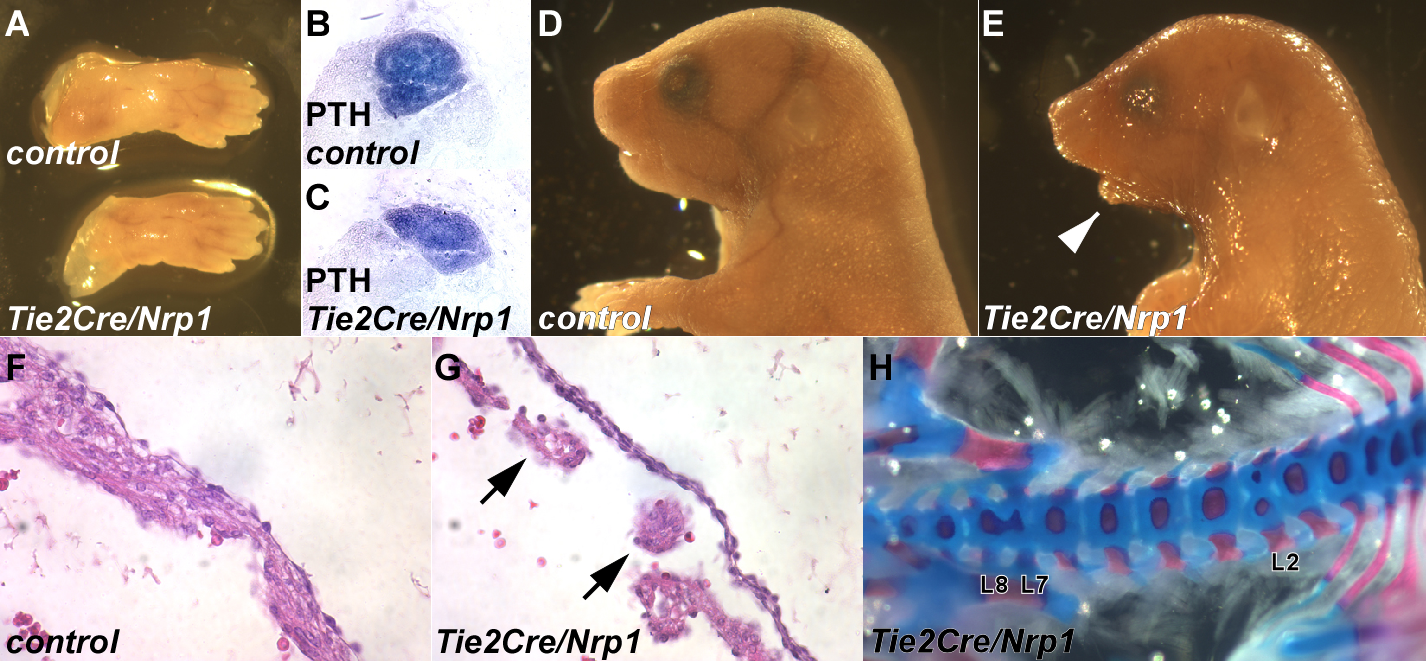

Supplement: Figure S1 — Embryogenesis in Tie2Cre/Nrp1 mutants. A, Comparison of E14.5 forelimbs from a control (top) and Tie2Cre/Nrp1 mutant (below) littermates to show normal limb development. B,C, In situ hybridization detection of parathyroid hormone to visualize the parathyroids in a control (A) and mutant (B) embryo at E18. D,E, Images of E14.5 heads of control (C) and mutant (D) embryos; the arrowhead in E points to the one obvious example of mandibular hypoplasia seen in this mutant background in this study. The shiny appearance of the embryo in panel E is a lighting artifact. F,G, Atrial myocardium at E14.5 in a control (F) and mutant (G) embryo; in the mutant, the myocardium is detached from the epicardium and is split into multiple pieces (two shown by arrows). H, Skeletal preparation of one mutant embryo at E18.5 showing vertebral defects, specifically an incompletely formed lumbar L2 vertebral body, and a partial fusion of the L7 and L8 vertebrae. (TIF) [file pone.0032429.s001.tif]

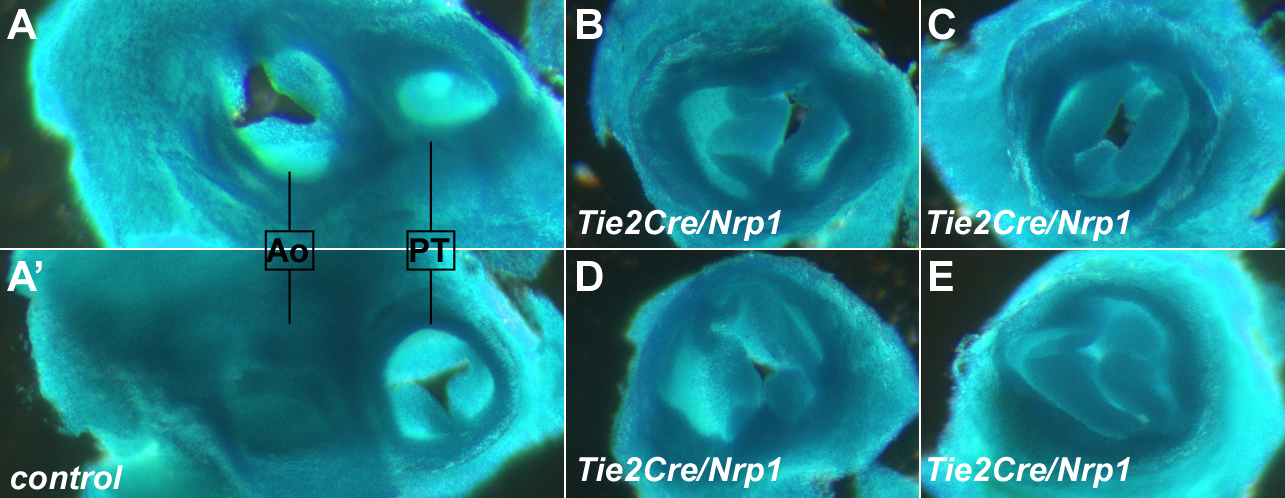

Supplement: Figure S2 — Visualization of valve leaflets at E18.5. A, A′, A single control embryo shown from two slightly different angles to visualize the aortic and pulmonary valves, each consisting of three well-formed leaflets. B–E, Four different mutants all showing a single outflow structure (i.e., a common arterial trunk) with a variety of valve leaflet morphologies and organizations. (TIF) [file pone.0032429.s002.tif]

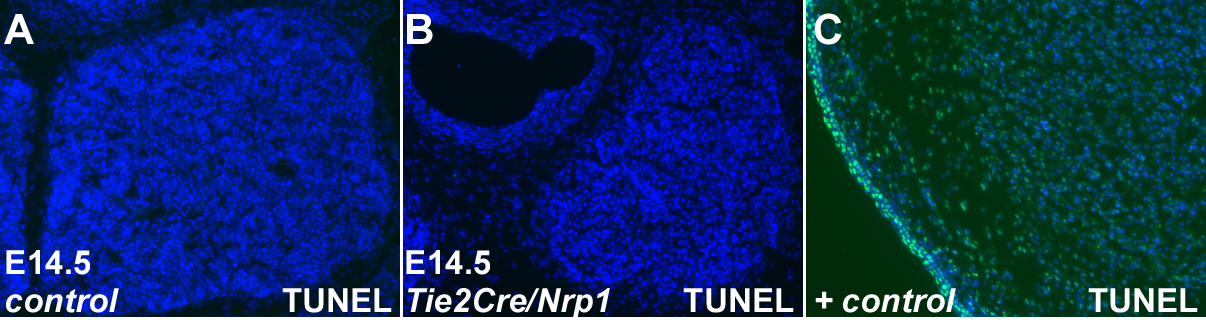

Supplement: Figure S3 — Absence of apoptosis in the developing thymus. A,B, Sections of E14.5 embryos stained by TUNEL labeling. Nuclei are counterstained in blue, and TUNEL-positive cells are in green; there were virtually no positive cells in either genotype. C, A positive control for staining is shown at the same magnification. (TIF) [file pone.0032429.s003.tif]

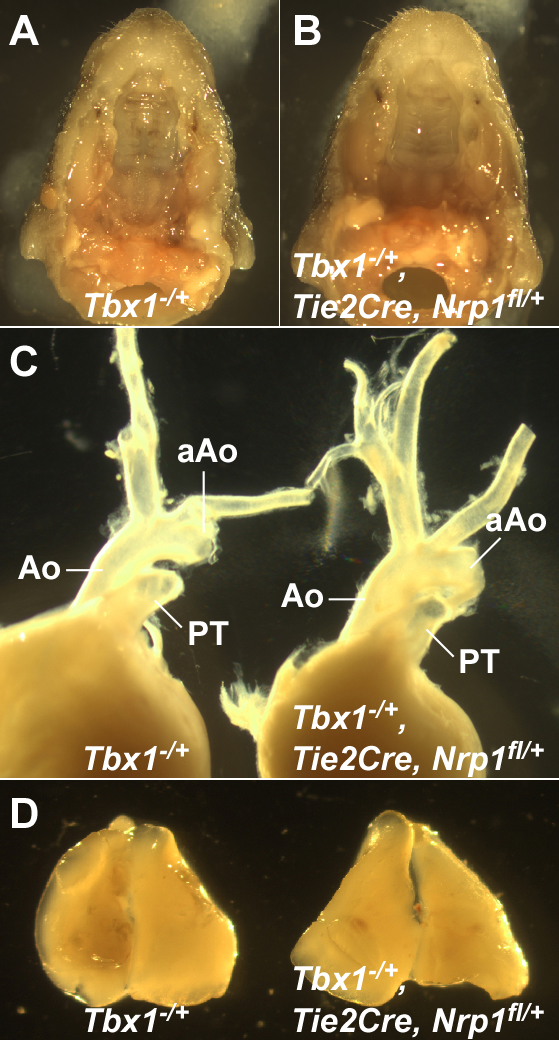

Supplement: Figure S4 — No accentuated phenotypes in Tbx1 - Nrp1 trans heterozygotes, all analyzed at newborn stage. A,B, Palates were fully closed. C, Heart outflow septation and great vessels were normal. D, Thymic size was normal. (TIF) [file pone.0032429.s004.tif]
